# Study of the Anatomical Association between Morton’s Neuroma and the Space Inferior to the Deep Transverse Metatarsal Ligament Using Ultrasound

**DOI:** 10.3390/diagnostics12061367

**Published:** 2022-06-01

**Authors:** María del Mar Ruiz-Herrera, Juan José Criado-Álvarez, Mario Suarez-Ortiz, Marko Konschake, Simone Moroni, Félix Marcos-Tejedor

**Affiliations:** 1 María del Mar Ruiz Clinic, 13600 Alcázar de San Juan, Spain; clinicamariadelmarruiz@gmail.com; 2 Department of Medical Sciences, Faculty of Health Sciences, University of Castilla-La Mancha, 45600 Talavera de la Reina, Spain; felix.marcostejedor@uclm.es; 3 Department of Health, Institute of Health Sciences, 45600 Talavera de la Reina, Spain; 4 Podosalud Clinic, 28011 Madrid, Spain; mario.suarez@podosaludclinica.es; 5 Department of Anatomy, Histology and Embryology, Institute of Clinical and Functional Anatomy, Medical University of Innsbruck (MUI), 6020 Innsbruck, Austria; marko.konschake@i-med.ac.at; 6 Department of Podiatry, Faculty of Health Sciences at Manresa, University of Vic—Central University of Catalunya (UVic-Ucc), 08500 Vic, Spain; dott.simonemoroni@gmail.com

**Keywords:** Morton’s neuroma, deep transverse metatarsal ligament, ultrasonography, podiatry

## Abstract

Morton’s neuroma (MN) is a common condition in clinical practice. The compressive etiology is the most accepted, in which compression occurs in the tunnel formed by the adjacent metatarsals, the deep transverse metatarsal ligament (DTML) and the plantar skin. Ultrasound (US) is a reliable method of study. The presence of insufficient space under the DTML may be related to the appearance of MN. Objectives: To verify the relationship between MN and the space under the DTML between the metatarsal heads of the third (M3) and the fourth (M4) metatarsals using US. Methods: This is a cross-sectional epidemiological study. The research study using the ultrasound (US) technique was carried out on 200 feet belonging to 100 patients aged 18 to 65 of both sexes, with a control group formed by 62 patients and a study group formed by 38 patients diagnosed with MN. Results: The presence of MN and the factors associated with it were studied in 100 patients using ultrasound (US). The assessment and comparison with US of the space inferior to the DTML between M3 and M4 in control groups and patients with MN show that patients with MN have a smaller size in the variable “h” (height or distance DTML-plantar skin), in the variable “b” (base or intermetatarsal distance M3 and M4) and in the variable “s” (surface of the parallelogram “h” × “b”). The predictors of MN are a decrease in dimension “b” and an increase in weight. Sitting in an office chair and the use of a bicycle, due to equinus, have an influence on the space below the DTML, reducing it and promoting the appearance of MN. Conclusions: The two US measurements (“h” and “b”) in the space below the DTML are smaller in patients with MN than in the asymptomatic group. A shorter distance between M3 and M4, and an increase in BMI are predictors of MN.

## 1. Introduction

Morton’s neuroma (MN) is a frequent condition in the general population with a prevalence of 30–33% and a female to male incidence of 4:1 [1]. Histological exams revealed no differences between resected MN and all the other common digital nerves in subjects without pain. It has been postulated that, instead of a benign tumor, MN pain is originated by neuropathic pain produced by an external compression of the third common digital nerve at the third web space.

It has been seen that there are several risk factors related to MN such as functional and biomechanical features, associated deformities and anatomical variations [2].

Clinically, patients complain of metatarsalgia, shooting and burning pain irradiating to the third and/or fourth toes. Pain worsens when wearing narrow or high-heeled shoes, leading, respectively, to a tighter intermetatarsal space, to increased plantar reaction forces and, finally, to an extension of the metatarsophalangeal joint, which results in compression of the common digital plantar nerve below the deep transverse metatarsal ligament (DTML) [3]. It has been hypothesized to be the result of the compression of the interdigital nerve as it passes under the DTML. It has also been suggested that the digital nerve is trapped between the third and fourth metatarsal heads, causing MN [4].

Although the cause of MN has not been clearly established, there are several theories and studies on its etiology. The compressive theory is the most common, according to which compression occurs in the tunnel formed by the two adjacent metatarsals, the DTML and the ground surface, which in weight-bearing conditions compresses the neurovascular bundle and generates the MN. This mechanical entrapment of the surrounding soft tissues has been suggested as the cause of the secondary changes that occur at the common plantar digital nerve and affect the formation of MN [4,5].

**Figure 1:**
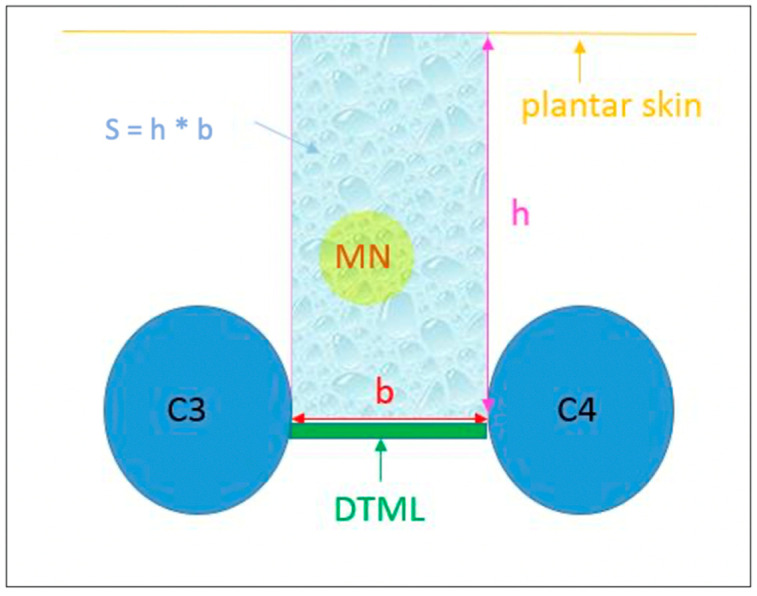
Representation of the anatomical space between the third and fourth metatarsal heads. C3 and C4: metatarsal heads 3 and 4; h: distance between the DTML and the plantar skin in the middle area between the M3 and M4 metatarsal heads; b: closest intermetatarsal distance between C3 and C4; MN in yellow circle: Morton’s neuroma.

There are studies which link this compression with the development of MN, and, on the other hand, there are studies that describe the diagnosis of NM through the use of US, as well as the ultrasonic anatomical description [1,4,6,7,8,9,10,11]. Nevertheless, none has measured the anatomical space below the DTML. Therefore, it is necessary to evaluate the anatomy of the surrounding structures related to MN, including the DTML (Figure 1).

Therefore, the purpose of this study is to use ultrasonography (US) to evaluate the anatomical relationship between MN and the DTML between the third (M3) and fourth (M4) metatarsal heads to evidence its correlation as one of the main predisposing factors for the development of MN.

## 2. Materials and Methods

This study has been designed as a cross-sectional descriptive epidemiological study, which was carried out between February 2020 and October 2020 in 100 patients (200 measurements) from Maria del Mar clinic (Alcazar de San Juan, Ciudad Real, Spain).

This study complies with the Declaration of Helsinki and received a favorable opinion from the Clinical Research Ethics Committee of the Universidad Rey Juan Carlos (Alcorcón, Madrid) under the registration number 1312201900320. Participants signed an informed consent form and provided demographical information.

### 2.1. Subjects

A total of 100 subjects (200 measurements) were included, who were evaluated by a single trained and qualified podiatrist (MM.R-H). The participants were between 18 and 65 years old, of legal age and had an active life [11]. These were divided into a control group formed by 62 patients who came to the clinic presenting other foot and ankle conditions not related to the study; and an MN study group formed by 38 patients who came to the clinic presenting symptoms compatible with MN. All participants were evaluated by the same podiatrist.

**Figure 2:**
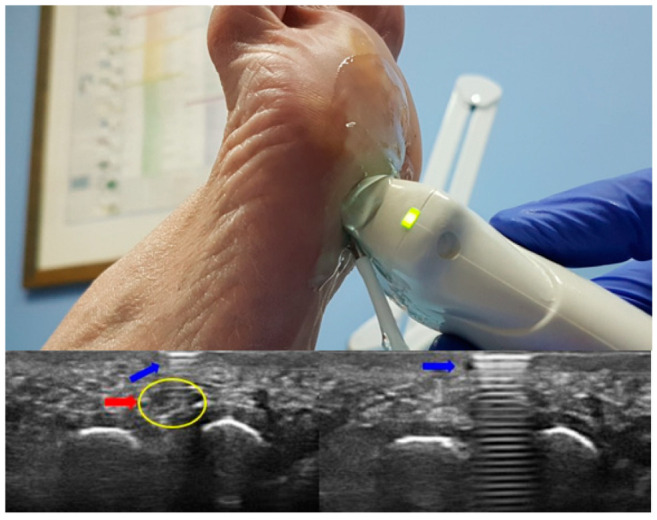
Painful echopalpation of MN: the tip of the pen presses on Morton’s neuroma, causing pain. The ultrasound image shows “the comet’s tail” produced by the tip of the pen. The blue arrow indicates the hyperechoic image produced by the pen, and the red arrow indicates the position of the MN. The yellow circle shows the MN.

The inclusion criteria for the MN group were neuropathic pain in the third IM space, positive US guided Mulder sign [11], positive US guided palpation [12] (Figure 2) and irradiating pain to the third and fourth toes [5].

Exclusion criteria for the MN group and control group were: subjects without solution of continuity of the plantar skin, presence of ulcers and/or vesicles, patients who had undergone surgery on the affected foot, congenital or acquired malformations, pregnant women, the presence of a keratotic lesion in the plantar area, physical exercise 48 h before examination, use of high heels 48 h before examination, equinus deformity, biomechanical alterations in lower extremities, no ultrasound visualization of the structures under study, alterations of the metatarsal parabola and rheumatic diseases [4,13,14].

### 2.2. Procedure and Intruments

The US scan was performed with a color Doppler ultrasound machine Canon “Aplio a” CUS-AA000 with linear probe transducer PLT-1005BT (5–15 MHz).

Dynamic US maneuvers provide reliable, functional data and have been shown to be accurate for diagnosis [15].

The anatomical US measurements below the DTML followed the same examination protocol. A preliminary examination protocol was carried out with the US, in which the probe was pressed on the plantar area both in the transverse position or short axis and in the longitudinal position or long axis to locate the DTML [6].

With this maneuver, the M3 and M4 heads are spread, showing the DTML between them as a hyperechoic band that pushes the tissues and/or the MN (if any) from the dorsal area to the plantar area of the foot. In the same way, while the evaluator pressed on the plantar area, he executed the Mulder’s maneuver, which consists of pressing the probe on the plantar area while compressing the frontal plane with the other hand on the heads of the first and fifth metatarsals, narrowing the third web space. With these two maneuvers, we can tense and relax the DTML to better locate it.

Once the DTML and the M3 and M4 heads are located, the exploration to get the measurements below the DTML will give us the image, which was taken with the probe perpendicular to the skin without touching it.

**Figure 3:**
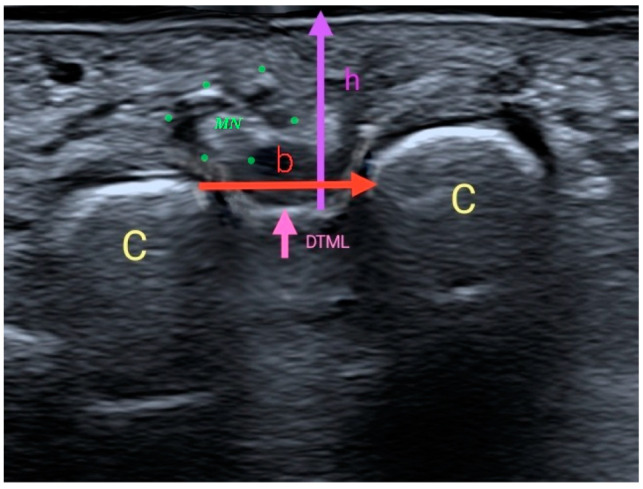
Ultrasound imaging of the measurements.C3 and C4: metatarsal heads 3 and 4 (M3 and M4); DTML: deep transverse metatarsal ligament; h: height: distance between the DTML and the plantar skin in the middle area between M3 and M4; b: base: distance between M3 and M4; MN: Morton’s neuroma.

Two measurements were taken with US [16]: a vertical measurement or height (h) between the plantar skin and the DTML in millimeters; and a horizontal measurement or base (b) between the area closer to the M3 and M4 heads. With these two measurements, a rectangular area was obtained (“s” = ”b” × “h”) in square millimeters (Figure 3).

In addition, other variables that could affect the anatomical US measurements were analyzed, such as weight, height, European shoe size, high impact physical activity, cycling hours, sitting hours (office chair), type of footwear, etc.

### 2.3. Analysis

The statistical analysis was conducted using SPSS 19.0 for Windows. In the descriptive statistical analysis, the parameters used depended on the variable of study. The Shapiro–Wilk test was used to determine variable distribution. The Bonferroni method was used to adjust multiple responses after the ANOVA test. The inferential statistical analysis of independent variables depended on the scale of each variable [17]. A type I error of 5% was considered [18].

## 3. Results

In 22 patients, an ultrasound visualization of the structures under study was not achieved, and finally a total of 200 feet of 100 patients were studied, of which 33 (33%) were male, 67 (67%) female, the mean age was 45.6 years (SD: 10.40; Median: 44.0; Range: 19–64), 62 (62%) were controls and 38 (38%) were MN patients (Table 1).

Studying the ultrasound measurements of b, h and s in men and women, according to the type of patient, we observed that there are statistically significant differences (*p* < 0.05) in all variables (Table 2). In general, b, h and s values are higher in controls than in MN patients.

The mean h on the right side was 12.4 mm. In the controls, the h on the right side was 13.0 mm, while in patients with MN it was 11.4 mm, a difference of 1.60 mm (*p* < 0.05). The mean h on the left side was 12.2 mm, with statistically significant differences according to type of patient (*p* < 0.05), with a difference of 2.0 mm given that controls had a mean of 13.0 mm (*p* < 0.05).

As for b, it had a mean size of 4.48 mm on the right side with a difference of 1.55 less in the MN group, whereas on the left side it was 4.46 mm with a difference of 1.58 mm, with statistically significant differences according to the type of patient (*p* < 0.05): smaller in the MN group than in the control group, and also smaller in the left foot than in the right foot.

Regarding sitting in an office chair (Table 3), there were statistically significant differences (*p* < 0.05) between men and women, with practically twice as many sitting hours in MN patients as in the controls. There was a difference of 30 h in women, and of 25.9 h in men.

The multivariate analysis using logistic regression, where the dependent variable is the type of patient (control and patient with MN), showed that the variables that were statistically significant (*p* < 0.05) were b (right and left) of the metatarsals and patient’s weight (Table 4).

## 4. Discussion

The measurements evaluated show that the dimensions were larger in the control group in both feet and smaller in patients with MN. Regarding “s”, since this is a linear combination of both dimensions (area of the parallelogram formed by “b” and “h”), it was reasonable to find that it was larger in the control group than in the MN group.

We can see that not only was h lower in the MN group than in the control group, but the MN group also had a lower h on the left side than on the right side. This left laterality also appears in the other variables and can be due to equinus.

The same is true of the surface. This may be in part due to the sitting position in office chairs. It has been observed that the left foot rests backwards on the toes in equinus position. Moreover, when we bend down, we usually place weight on the dominant side, i.e., the right side, resting the left knee on the floor and keeping the left foot in equinus with a forced dorsal flexion of the toes. This produces compression on the metatarsal area and reduces both h and b space.

A previous study [13] evaluated this anatomical region using magnetic resonance imaging (MRI) and measured the DTML thickness and the IM area of the second and third spaces. They found a surface of 56.6 in the control group (SD 14.66) between M3 and M4, and of 52.8 (SD 15.12) in the MN group; so this surface was smaller in the MN group. Our results are similar: although we measured a different area, this encompasses the same space, and the surface is smaller in the MN group compared to the control group in both studies. However, the anatomical region studied by Stecco et al. is different from ours: their IM space area is delimited dorsally by the DTML, plantarly by the plantar fascia, and laterally by the flexor tendons, whereas in this study, the space is delimited by the DTML, the closest area between M3 and M4, and the plantar skin.

Other studies have used US for the diagnosis and evaluation of NM, but none have metrically analyzed the anatomical structures related to NM [1,7,8,9,10,11]. In addition, our study adds the clinical advantages of using US [19], through a protocol that we evaluated in a previous work that obtained a concordance of 99.58%, thus guaranteeing the measurements [6]. All this guarantees that the evaluations of the new measurements (h, b) studied with US have been carefully verified, with the opinion of two evaluators. Other limitations of the MRI study, as against ours, are that it works with a smaller sample size (40 versus 200) and that it does not compare the right foot with the left foot. In both studies, with MRI and US, despite measuring a different area but encompassing the same space, the surface is smaller in the MN group compared with the control group.

Of the population studied, 55.3% of women and 44.7% of men presented MN, which is in line with the incidence of previous works, 75% and 55%, respectively [20]. This may be due to the most widespread habits according to sex, such as footwear used, sporting activity, or access to similar jobs in which sitting is very common [21]. According to this, our results could be due to the fact that women mostly use narrow shoes with high heels and thin soles. This is a predisposing factor for the development of MN because of the compression under the DTML and, reducing both “b” and “h” measurements, the greater compression on the forefoot due to the equinus position [22].

On the other hand, men do more hours of high-impact physical activity and of cycling (3.9 versus 1.5 h and 3.4 versus 1.8 h, respectively). This can be explained by the maintained anatomical position of the foot in equinus, which can develop compression neuropathies, as described in previous works [2,23,24].

Weight is a variable that influences the development of MN [25,26], which explains the fact that, in our study, weight and “b” measurement are strong predictors of MN in both sexes. From a point of view of prediction, on the whole, a higher weight and a smaller “b” measurement are good predictors of MN. The logistic regression multivariate analysis where the dependent variable is the type of patient (control and MN patient) has shown that the statistically significant variables (*p* < 0.05) have been the patient’s weight and the bases (right and left) of the metatarsals.

As for the metatarsals bases, our results show that the larger the size of the base, the lower the risk of MN. This is taken to mean that for each one unit increase in the size of the base, the risk of MN is reduced 0.054 for the left side and 0.36 for the right side. This is consistent with studies about the use of narrow footwear as a trigger for forefoot conditions and for MN [22,27] because narrow shoes decrease the IM distance, compressing the tissues and therefore reducing the b distance.

Furthermore, structural equinus were excluded from this study to demonstrate that the development of MN in the study population was caused by equinus resulting from habits, as previous works have described as a predisposing factor, because the equinus produces compression on the forefoot due to the Achilles-calcaneal-plantar system [2,4,28], thus evidencing the predisposition to develop MN as a result of the foot’s equinus position when cycling or sitting in office chairs.

At the diagnostic level, the latest consensus document of the European Society of Radiology (2017) established US as the technique of choice for the diagnosis of MN [29], which points out the value of our results. Thus, US gained clinical importance in the approach to MN and is an asset in its surgical decompression. To do this, most surgical techniques aim at increasing the space under the DTML. Though this attempt to create more space for the MN by cutting the DTML is a very common technique, no publication has been found that measures this space and that connects it with MN. For this reason, our results may be reinforced amidst the great variety of surgical techniques which aim at freeing the DTML to create more space for the MN [30,31,32,33,34,35,36].

Finally, it should be pointed out that one of the possible limitations of this study is that during the dynamic ultrasound evaluation, it is necessary to avoid involuntary movements of the transducer on the skin because this could bias the results as described in the literature [37]. It must be borne in mind that the use of US requires a learning curve and that the evaluators must be experienced professionals, as the visualization of the IM space is complex and can lead to mistakes. Added to this, some patients are poor conductors of US.

It would be interesting to extend the measurements on a large sample to identify if an S value that could be a predictable factor of NM and including other variables which might be connected with the condition under study. Likewise, it would also be interesting to include issues such as how long the patient has been suffering MN, to see if a longer duration of the condition may increase (or reduce) the space.

We propose to extend and confirm this study, together with the variables that seem to be predictors of MN. The pressure placed by the pedal of the bicycle on a small area of the forefoot, as well as the unnatural equinus position of the foot when sitting in office chairs for long hours would be an interesting subject of study. It would be feasible to design chairs which keep the foot from moving back or from resting on the bars of the wheels, making the foot remain resting fully on the floor at an angle of 90 degrees while sitting.

## 5. Conclusions

In conclusion, the two US measurements performed (h and b) as objectives of this study in the space below the DTML, are smaller in patients with MN than in the asymptomatic group. The shorter distance between M3 and M4, and the increase in BMI are predictors of MN.

## Figures and Tables

**Table 1:** Description of the study population.

		Sexo		
		Men	Women	Total
**Group**	**Control**	16 (25.8%)	46 (74.2%)	62
	**Case**	17 (44.7%)	21 (55.3%)	38
	**Total**	33 (33.0%)	67 (67.0%)	100

**Table 2:** Results according to type of patient, sex and ultrasound measurements (SD: standard deviation). * statistically significant.

Sex	Variable	Group		Statistical Significance
		Control	Case	
**Men**	**right height (mm)**	16.4 (SD: 2.28)	12.6 (SD: 1.77)	*p* < 0.05 *
	**right base (mm)**	6.4 (SD: 1.41)	3.6 (SD: 0.76)	*p* < 0.05 *
	**right surface (mm^2^)**	108.1 (SD: 34.84)	45.3 (SD: 9.27)	*p* < 0.05 *
	**left height (mm)**	16.5 (SD: 2.12)	12.5 (SD: 1.77)	*p* < 0.05 *
	**left base (mm)**	6.5 (SD: 1.37)	3.6 (SD: 0.60)	*p* < 0.05 *
	**left surface (mm^2^)**	109.5 (SD: 34.55)	44.7 (SD: 6.64)	*p* < 0.05 *
**Women**	**right height (mm)**	11.8 (SD: 1.14)	10.4 (SD: 1.38)	*p* < 0.05 *
	**right base (mm)**	4.6 (SD: 0.45)	3.4 (SD: 1.00)	*p* < 0.05 *
	**right surface (mm^2^)**	54.6 (SD: 10.40)	36.8 (SD: 15.51)	*p* < 0.05 *
	**left height (mm)**	11.8 (SD: 1.13)	9.6 (SD: 0.99)	*p* < 0.05 *
	**left base (mm)**	4.5 (SD: 0.45)	3.3 (SD: 0.66)	*p* < 0.05 *
	**left surface (mm^2^)**	54.2 (SD: 10.54)	33.0 (SD: 9.91)	*p* < 0.05 *

**Table 3:** Results according to type of patient, sex, physical activity and sitting position. (SD: Standard deviation). * statistically significant.

Sex	Variable	Group		Statistical Significance
		Control	Case	
**Men**	**Sitting (hours)**	25.4 (SD: 16.86)	51.3 (SD: 18.34)	*p* < 0.05 *
	**Bicycle (hours)**	2.8 (SD: 2.70)	4.0 (SD: 4.28)	*p* > 0.05
	**Exercise (hours)**	4.4 (SD: 4.36)	3.4 (SD: 3.41)	*p* > 0.05
**Woman**	**Sitting (hours)**	25.2 (SD: 11.12)	55.2 (SD: 16.97)	*p* < 0.05 *
	**Bicycle (hours)**	0.9 (SD: 1.50)	3.6 (SD: 5.64)	*p* < 0.05 *
	**Exercise (hours)**	1.5 (SD: 2.32)	1.4 (SD: 1.88)	*p* > 0.05

**Table 4:** Logistic regression model: B: beta coefficient; Exp(B): beta exponential; 95% CI: 95% confidence interval.

Variables	B	Statistical Significance	Exp(B)	95%CI for Exp(B)	
				Lower	Upper
**Weight**	0.07	0.018	1.073	1.012	1.137
**Left Base**	−2.916	0.000	0.054	0.013	0.225
**Right Base**	−1.011	0.021	0.364	0.154	0.857
**Constant**	10.206	0.004	27,061.890		
